# Exogenous 2-Keto-L-Gulonic Acid Supplementation Promotes L-Ascorbic Acid Biosynthesis in *Drosophila melanogaster*

**DOI:** 10.3390/ijms27020978

**Published:** 2026-01-19

**Authors:** Chuxiong Meng, Hui Xu

**Affiliations:** 1 Department of Chemistry, University of Wisconsin-Madison, Madison, WI 53706, USA; cmeng35@wisc.edu; 2 Institute of Applied Ecology, Chinese Academy of Sciences, Shenyang 110016, China

**Keywords:** L-ascorbic acid (ASA), 2-keto-L-gulonic acid (2KGA), *Drosophila melanogaster*, biosynthesis, antioxidant defense, metabolic regulation, animal nutrition

## Abstract

L-ascorbic acid (ASA) is an essential micronutrient critical for antioxidant defense and metabolic regulation in animals. Unlike many vertebrates, *Drosophila melanogaster* possesses the ability to synthesize ASA endogenously, yet the regulatory mechanisms governing this biosynthesis remain unclear. 2-keto-L-gulonic acid (2KGA), a key precursor in industrial vitamin C production, has been shown to enhance ASA accumulation in plants, but its role in invertebrates is unknown. This study systematically investigated the effect of exogenous 2KGA supplementation on ASA biosynthesis in *Drosophila*. Fruit flies were reared on media with or without 2KGA (1.6 g/L; *n* = 30 per group) for 12 days, followed by ASA quantification via high-performance liquid chromatography (HPLC). Results showed that 2KGA treatment increased mean ASA content from 0.00853 ± 0.0012 to 0.01064 ± 0.0015 μg/fly (24.74% increase; *p* = 0.0194, *r*^2^ = 0.558) compared to the control group, indicating that 2KGA acts as a metabolic regulator to promote ASA biosynthesis. Sex-separated analyses further revealed that this effect was primarily driven by male flies (*p* = 0.0057, *r*^2^ = 0.879), whereas females showed no significant response (*p* = 0.1783), pointing to a sex-dependent regulation of 2KGA-mediated ASA biosynthesis. These findings provide the first evidence that 2KGA modulates ASA levels in an invertebrate disease model and suggest that fruit flies can serve as a useful platform to explore conserved redox-regulatory mechanisms relevant to human health and disease.

## 1. Introduction

L-ascorbic acid (ASA), a vital micronutrient, plays a fundamental role in antioxidant defense and metabolic regulation across diverse animal taxa [1]. Its unique ability to undergo sequential one-electron oxidations—generating monodehydroascorbate and dehydroascorbate—enables ASA to function as both a cofactor and a potent antioxidant [2]. Accumulating evidence highlights its critical roles in promoting growth, modulating immune responses, and enhancing resistance to environmental stressors and diseases in animals [3,4,5].

While ASA is ubiquitously present in plants and vertebrates, its biosynthesis exhibits striking taxonomic variability: all plant species investigated to date possess endogenous ASA synthetic pathways [6], whereas certain vertebrates—including bats, birds, primates (e.g., humans), and bony fish—have lost this capacity due to genetic inactivation, necessitating dietary intake [6].

In contrast to plants and vertebrates, our understanding of ascorbic acid biosynthesis in invertebrates remains limited. The shared absence of L-gulonolactone oxidase (GULO) gene has led to the hypothesis that invertebrates, akin to humans and other primates, may lack functional ASA biosynthetic pathways [7]. Notably, several studies have demonstrated that fruit flies can maintain appreciable levels of L-ascorbic acid even when reared for multiple generations on vitamin C–deficient diets, indicating that diet is not the sole source of vitamin C synthesis in fruit flies [8]. Massie et al. reported that whole-body L-ascorbic acid levels in *D. melanogaster* decline with age but transiently increase after brief cold shock, indicating the existence of internal regulatory routes that modulate ASA biosynthesis [9]. Subsequent work by Henriques et al. excluded a major contribution of the gut microbiota by showing that axenic flies maintain similar ASA levels to conventionally reared controls and that ex vivo cultures of the microbiome do not accumulate vitamin C [10]. In the same study, flies exposed to 15 °C for one day exhibited a decrease in ASA content, which returned to baseline after one day of recovery at 25 °C, demonstrating that ASA metabolism in *Drosophila* can actively respond to environmental temperature changes [10]. Together, these findings indicate that, despite the apparent absence of a canonical GULO gene, *D. melanogaster* is capable of synthesizing vitamin C via as-yet-uncharacterized biosynthetic pathways. Given ASA’s conserved role in physiological homeostasis, elucidating its biosynthetic pathways in understudied invertebrate models and developing strategies to enhance ASA content remain scientifically imperative.

2-keto-L-gulonic acid (2KGA), a key intermediate in industrial vitamin C production, is typically generated via a two-stage microbial fermentation involving *Gluconobacter oxydans*, *Ketogulonicigenium vulgare*, and *Bacillus megaterium* [11,12,13]. During crystallization, this process yields a by-product (residue after evaporation, RAE) containing ~25% 2KGA alongside formic acid, oxalic acid, proteins, nucleic acids, and water [14]. Previous studies have demonstrated that RAE promotes plant growth and ASA accumulation: in *Brassica campestris* L., for example, RAE supplementation increased biomass by 23.9% and ASA content by 188.94% [15]. Gao et al. [16] further showed that exogenous 2KGA enhances ASA biosynthesis in plants by upregulating key enzymes, thereby mitigating salt stress. Stable isotope tracing has confirmed 2KGA’s role as a direct precursor in plant ASA biosynthesis [16], while studies in zebrafish [17] have extended this mechanism to vertebrates, demonstrating that 2KGA supplementation elevates ASA levels comparable to direct ASA administration.

**Figure 1:**
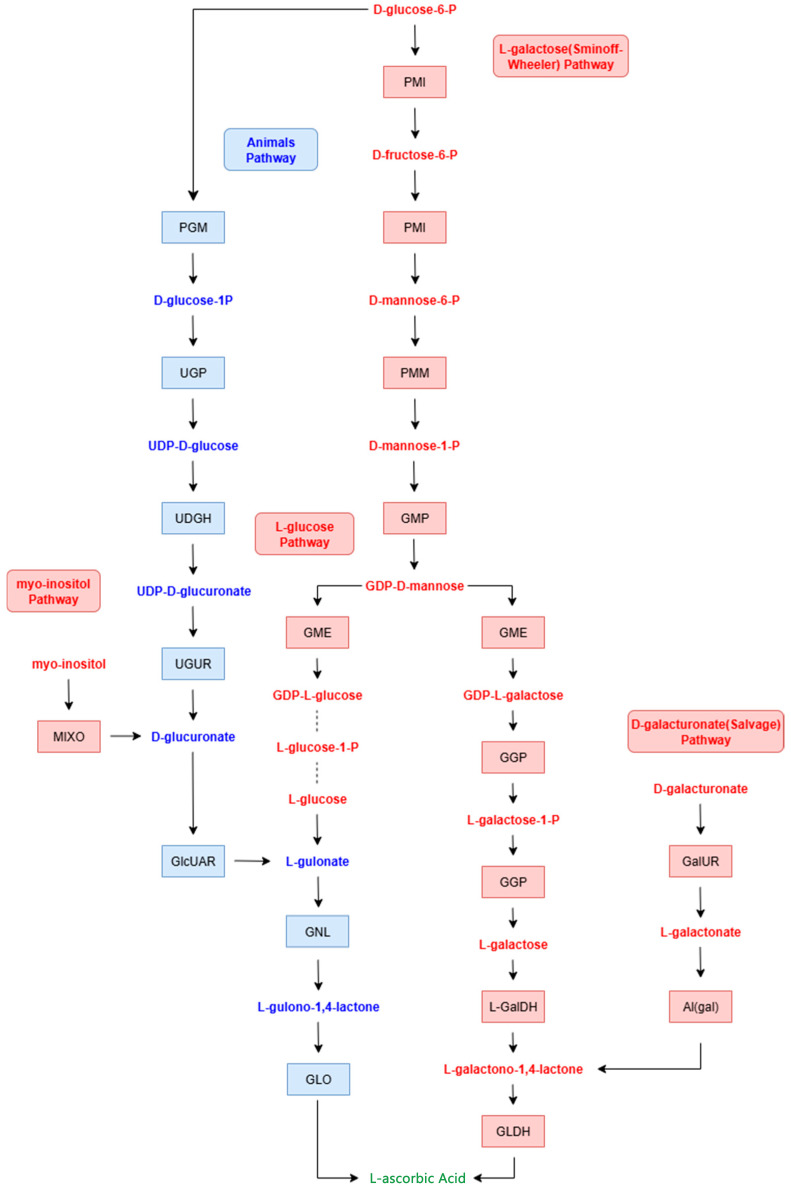
Biosynthetic pathways of ascorbic acid in plants and animals. The diagram illustrates the metabolic routes for ascorbic acid (L-ascorbic acid) synthesis in plants and animals. Key pathways are highlighted in blue, indicating the primary routes. The figure includes enzymatic steps and intermediates: L-galactose pathway (plants), involving enzymes such as GDP-mannose pyrophosphorylase and L-galactose dehydrogenase; and animal pathway (via glucuronate), involving enzymes like UDP-glucuronate pyrophosphorylase and L-gulonolactone oxidase. Additional pathways and enzymes, such as myo-inositol and D-glucuronate, are also depicted. Abbreviations: GLD, L-gulonolactone dehydrogenase; ASA, ascorbic acid.

The conserved ability of 2KGA to enhance ASA biosynthesis across plants and vertebrates may stem from the shared terminal step of ASA synthesis: in most animals, L-gulono-1,4-lactone is converted to ASA by L-gulono-1,4-lactone oxidase (GLO) (Figure 1), a mechanism identical to the L-gulose pathway in plants [18,19,20,21,22]. However, whether invertebrates employ analogous pathways—or possess GLO-like enzymatic activity—remains unknown. Here, we hypothesized that 2KGA could serve as a metabolic precursor to enhance ASA biosynthesis in *Drosophila melanogaster*, an established invertebrate model for genetic and metabolic research [23]. Testing this hypothesis would not only expand the known taxonomic scope of 2KGA’s role in ASA biosynthesis but also provide mechanistic insights into de novo ASA synthesis in invertebrates, with implications for animal nutrition and stress resilience. Importantly, *Drosophila melanogaster* is widely used as a classical model organism to study human conditions, including aging, neurodegeneration, metabolic disorders, and oxidative stress–related pathologies. Although humans lack the ability to synthesize L-ascorbic acid endogenously, the fundamental cellular roles of ASA in redox homeostasis are highly conserved across species [16]. Therefore, investigating how exogenous metabolic precursors regulate ASA levels in *Drosophila*-based disease and stress models may provide valuable mechanistic insights relevant to human nutritional physiology and redox-related disorders.

## 2. Results

### 2.1. Effect of Exogenous 2-Keto-L-Gulonic Acid on the Total ASA Content of Drosophila

Following the extraction from homogenized *Drosophila melanogaster* (fruit fly) samples, the L-ascorbic acid (ASA) content was quantified using high-performance liquid chromatography (HPLC). The mean ASA content of fruit fly was 0.00853 ± 0.0012 µg/fruit fly in the negative control group (NC), 0.01064 ± 0.0015 µg/fruit fly in the 2-keto-L-gulonic acid (2KGA) treatment group (T), and 0.01883 ± 0.0021 µg/fruit fly in the positive control group (PC). Each group comprised 30 fruit flies (*n* = 30) distributed across three replicates, ensuring the reliability of the results.

**Figure 2:**
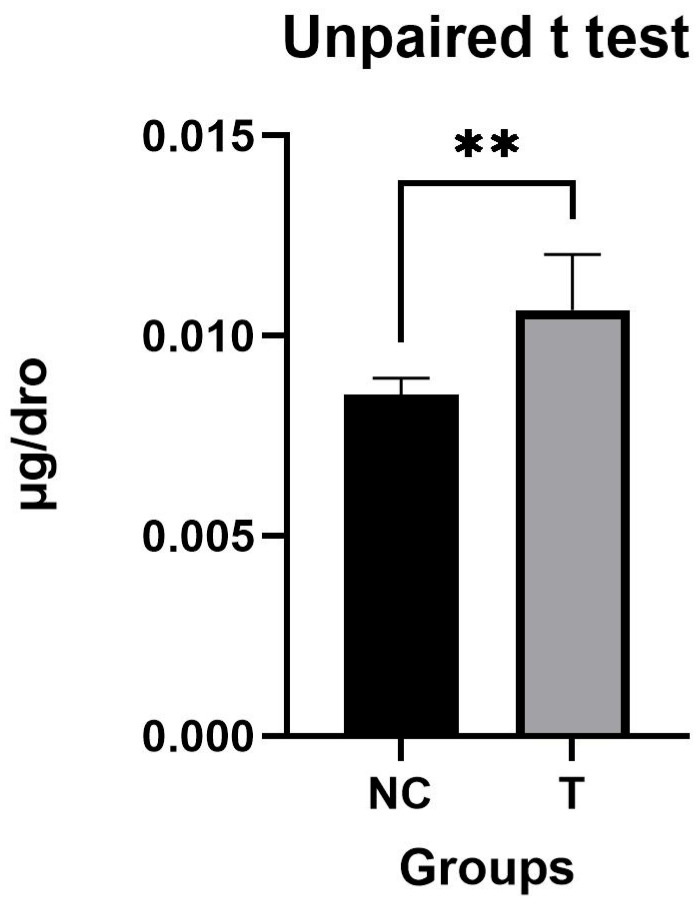
Effect of exogenous 2-keto-L-gulonic acid on the total ASA content of *drosophila*. NC, control; T,2-keto-L-gulonic acid. Number of asterisks corresponds to the significance level of *p*-values: more asterisks indicate greater significance. μg/dro represents the average ASA content per fruit fly.

**Figure 3:**
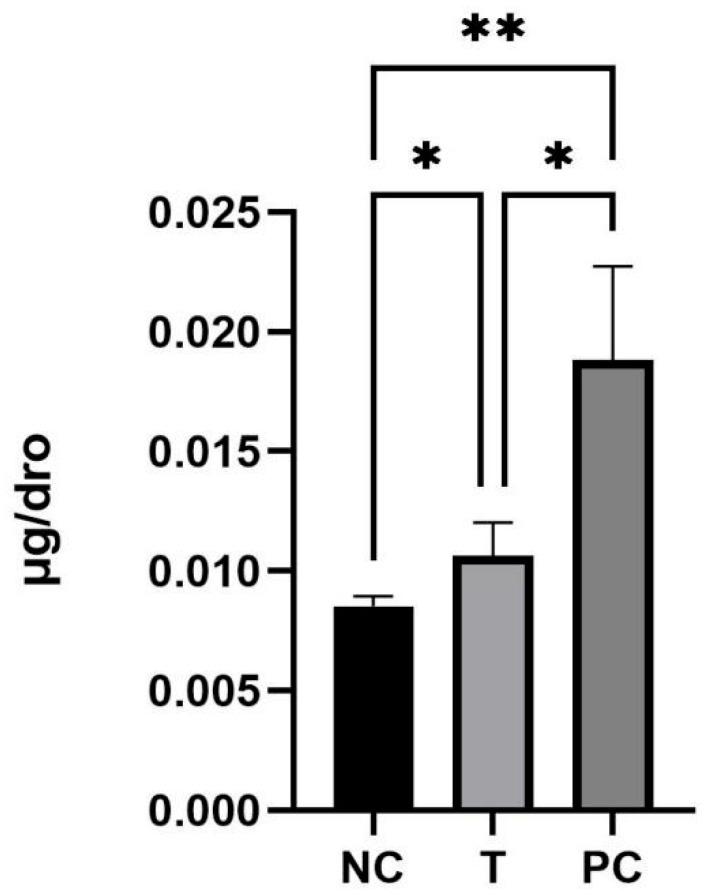
Effect of exogenous 2-keto-L-gulonic acid on the total ASA content of *drosophila*. NC, control; T,2-keto-L-gulonic acid; PC, L-ascorbic acid. Number of asterisks corresponds to the significance level of *p*-values: more asterisks indicate greater significance. μg/dro represents the average ASA content per fruit fly.

Statistical analysis revealed notable findings. A two-tailed unpaired *t*-test demonstrated that the ASA content in the 2KGA treatment group (T) was significantly higher than that in the negative control group (NC), with a *p*-value equal to 0.0194 and a coefficient of determination (*r*^2^) of 0.5582 (Figure 2). To comprehensively evaluate the differences among all three groups, a one-way analysis of variance (ANOVA) was conducted. The results of the one-way ANOVA indicated significant variations in ASA content across the groups (*p* < 0.01) (Figure 3). Subsequently, post hoc analysis further validated significant differences in ASA content between the NC and T groups (*p* < 0.05), as well as between the NC and PC groups (*p* < 0.01). However, no statistically significant difference was observed between the T and PC groups (*p* > 0.05), suggesting that the effect of 2KGA on ASA biosynthesis in *Drosophila melanogaster* was distinct from the direct supplementation of ASA but not equivalent to the full impact of ASA addition.

### 2.2. Effect of Exogenous 2-Keto-L-Gulonic Acid on the Total ASA Content of Sex-Controlled Drosophila

**Figure 4:**
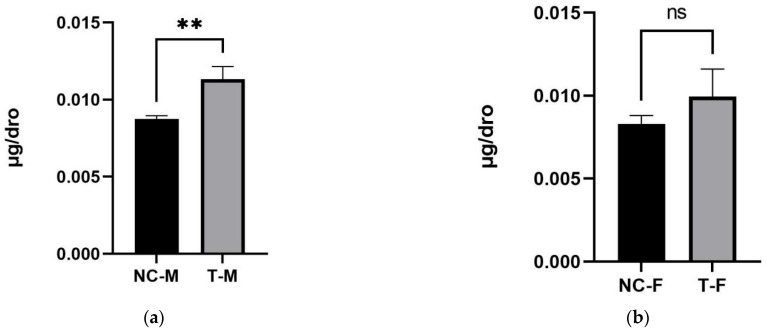
Effect of exogenous 2-keto-L-gulonic acid on the total ASA content in sex-controlled Drosophila: (**a**) total ASA content in male flies under control conditions (NC-M) and after 2-keto-L-gulonic acid supplementation (T-M); (**b**) total ASA content in female flies under control conditions (NC-F) and after 2-keto-L-gulonic acid supplementation (T-F). The number of asterisks indicates the significance level (*p* values), with more asterisks indicating greater significance; ns indicates no significant difference.

To further investigate the potential influence of sex on ASA biosynthesis, a sex-based comparison was conducted using two-tailed unpaired *t*-tests (Figure 4). The mean ASA content of female fruit fly was 0.00831 ± 0.0005 µg/fruit fly in the negative control group (NC-F), 0.00995 ± 0.0016 µg/fruit fly in the 2-keto-L-gulonic acid (2KGA) treatment group (T-F), and for male group, 0.01569 ± 0.0029 µg/fruit fly in the negative control group (NC-M), 0.01133 ± 0.0008 µg/fruit fly in the 2-keto-L-gulonic acid (2KGA) treatment group (T-M). Each group comprised 30 fruit flies (*n* = 30) distributed across three replicates, ensuring the reliability of the results. In female fruit flies, the ASA content did not differ significantly between the 2KGA treatment group and the negative control group (*p* = 0.1783, *r*^2^ = 0.3994). In contrast, male flies exhibited a significant increase in ASA content in response to 2KGA treatment compared to the negative control (*p* = 0.0057, *r*^2^ = 0.8788). These findings suggest a sex-dependent response to 2KGA, with male flies showing greater sensitivity in terms of ASA biosynthesis enhancement.

Taken together, these sex-separated analyses indicate that the increase in ASA observed in the mixed-sex experiments is driven primarily by male flies, while females show a weaker or absent response. This pattern points to a sex-dependent regulation of 2KGA-mediated ASA biosynthesis in *D. melanogaster*. A previous study by Henriques et al. also revealed pronounced sex-dependent differences in vitamin C levels in *Drosophila* [10]. Together with our findings, these prior observations support the notion that ASA metabolism in *D. melanogaster* is strongly sex-dependent and likely involves regulatory layers beyond simple differences in body size, although the precise biochemical and physiological mechanisms remain unclear.

It should be noted that the coefficient of determination for the mixed-sex comparison (*r*^2^ = 0.5582) indicates only a moderate proportion of variance in ASA levels being explained by 2KGA treatment. This is likely due to the fact that males and females were pooled in this analysis, which increases within-group variability because the two sexes differ in both baseline ASA levels and responsiveness to 2KGA. When the data were analysed separately by sex, the explanatory power of 2KGA increased markedly in males (*r*^2^ = 0.8788), indicating a robust and consistent treatment effect in this group that was partially obscured in the pooled analysis. In contrast, the relatively low *r*^2^ and non-significant *p*-value observed in females suggest that, under the present experimental conditions, we cannot clearly determine whether 2KGA exerts a measurable stimulatory effect on ASA biosynthesis in female flies, potentially due to additional physiological factors that modulate ASA homeostasis in this sex.

## 3. Discussion

Recent studies have increasingly spotlighted exogenous 2-keto-L-gulonic acid (2KGA) as a potent enhancer of L-ascorbic acid (ASA) biosynthesis. This phenomenon has been consistently observed across diverse plant species, including *Arabidopsis thaliana*, *Brassica rapa*, *Spinacia oleracea*, and *Capsicum annuum* [16]. These investigations have not only demonstrated that 2KGA supplementation significantly elevates ASA levels but also that it enhances plants’ resilience to various environmental stresses [16]. In addition, our laboratory’s previous research further extended this concept to aquatic organisms, showing that 2KGA administration led to a notable increase in ASA content in zebrafish, thus marking an initial foray into understanding its effects in animal models. Building upon these findings, the present study explored the impact of 2KGA on *Drosophila melanogaster*, an invertebrate model. Remarkably, the results revealed a 24.74% increase in ASA content compared to the control group. To the best of our knowledge, this constitutes the first evidence of 2KGA-mediated ASA enhancement in an invertebrate species, suggesting a potentially broad taxonomic applicability of 2KGA in modulating ASA biosynthesis.

The supplementation of 2KGA in *Drosophila melanogaster* resulted in a significant 24.74% increase in ASA content, indicating a considerable conversion efficiency of 2KGA to ASA within this organism. This finding aligns closely with previous research on plants and zebrafish, where 2KGA intake elevated ASA levels by 26.19% in *Arabidopsis thaliana*, 20.73% in *Brassica rapa*, and 25% in zebrafish [16]. These consistent results across plant and piscine species, and now invertebrates, underscore 2KGA’s potential as a reliable precursor in enhancing ASA biosynthetic pathways.

However, the present study also revealed a striking disparity in ASA content in fruit flies reared on ASA-supplemented media. Compared to the negative control group, the ASA content in the ASA-supplemented group increased by 120.75%. This disproportionately large increase is likely an experimental artifact, possibly due to direct adhesion of ASA from the culture medium to the fruit flies during rearing, which could have skewed the final measurement results. These findings highlight the need for refining extraction methods and emphasize the importance of considering potential sources of error in future studies. Additionally, the study’s limitations, including a relatively small sample size (*n* = 30 per group) and reliance on a single metric (ASA content), call for further research incorporating multiple parameters, such as enzymatic activity assays, to comprehensively understand the underlying mechanisms

It is widely recognized that many animal species synthesize ASA through the glucuronide pathway, initiating with D-glucose as the primary substrate. This biosynthetic process involves multiple enzymatic steps, wherein D-glucose is transformed into L-gulono-1,4-lactone through the action of regucalcin (also identified as senescence marker protein 30, SMP30) [24]. Subsequently, L-gulono-1,4-lactone undergoes a critical oxidation reaction catalyzed by L-gulono-1,4-lactone oxidase (GLO), leading to the production of ASA [25]. GLO serves as the rate-limiting enzyme in this final step of the ASA synthesis pathway in animals, playing a pivotal role in regulating the efficiency of this metabolic process [26]. In plant systems, the terminal phase of both the myo-inositol and L-gulose biosynthetic pathways involves the enzymatic action of GLO, facilitating the conversion of L-gulono-1,4-lactone to ASA, a mechanism analogous to that observed in animal models. Recent investigations by our group, supported by findings from Gao et al. [16], have indicated that exogenous 2KGA significantly promotes ASA accumulation in plants, with GLO identified as a critical enzyme in this process. These observations suggest that the L-gulose pathway may be substantially activated during the transformation of 2KGA to ASA. Extending this hypothesis to fruit flies, the observed 24.74% increase in ASA content following 2KGA supplementation could imply a conserved role for GLO or a related enzymatic system. Although previous studies have reported that invertebrates, teleost fish, certain passerine birds, and hominoids lack the GLO enzyme required to catalyze the final step of ASA synthesis, resulting in the loss of ASA biosynthetic capacity [7], recent findings have challenged this notion. Emerging evidence suggests that some chondrichthyan and non-teleost bony fish retain the ability to synthesize ASA. Furthermore, research by Shi et al. [17] has hypothesized that, under the influence of 2KGA, zebrafish may express isoenzymes with GLO-like activity. Extending this hypothesis to fruit flies, the observed increase 24.74% in ASA content following 2KGA supplementation could indicate a conserved role for GLO or a related enzymatic system, meriting further investigation to elucidate the potential metabolic regulation in this invertebrate species.

Beyond its relevance in basic insect physiology, the present findings may also be relevant to human health research. Humans have lost the ability to synthesize L-ascorbic acid because of mutations in the GLO gene, and therefore depend entirely on dietary intake to maintain redox balance and cellular stability. Oxidative stress caused by reduced antioxidant capacity is widely recognized as a major factor in aging and many human diseases, including cardiovascular diseases, neurodegenerative disorders, and cancer [27]. *Drosophila melanogaster* is a well-established model organism for studying these human conditions because of its conserved stress-response pathways and powerful genetic tools. Our observation that exogenous 2KGA increases endogenous ASA levels in *Drosophila* suggests that alternative or compensatory metabolic pathways may exist to enhance antioxidant capacity. These pathways may reflect conserved regulatory strategies and provide a useful basis for guiding future nutritional or redox-modulating strategies to improve redox balance in humans.

As a critical intermediate in the industrial production of L-ascorbic acid (ASA), 2-keto-L-gulonic acid (2KGA) holds significant practical value. The current industrial synthesis of ASA generates substantial volumes of waste liquid containing 2KGA, and the effective utilization of this 2KGA could substantially reduce waste treatment costs. Serving as a precursor in industrial ASA synthesis, 2KGA undergoes a complex process involving lactonization and enolization, among other chemical methods, to yield the final ASA product, a procedure that is both energy-intensive and costly [13]. Notably, the market price of 2KGA is approximately one-third that of ASA, coupled with its chemical stability, accessibility, and the absence of additional chemical conversion steps, rendering it a more cost-effective option for market applications [28,29]. Despite its potential, 2KGA has received limited industrial attention, not to mention its role in nature. Prior to our research on *Brassica rapa* and the current study on *Drosophila melanogaster*, only a single publication had explored 2KGA, noting its role in tartaric acid synthesis in plants [30]. Our study expands the understanding of 2KGA’s capacity to enhance ASA synthesis in an animal model, suggesting that further exploration of 2KGA’s role in augmenting biological ASA levels could open up new avenues for research and practical applications in both biological and industrial contexts.

## 4. Materials and Methods

To investigate the effect of exogenous 2-keto-L-Gulonic Acid (2KGA) on L-ascorbic acid (ASA) biosynthesis in *Drosophila melanogaster*, a controlled experiment was conducted. Using a quantitative approach, we compared ASA levels in fruit flies fed on media supplemented with 2KGA, ASA, or without supplementation (2KGA and ASA were provided by Northeast Pharmaceutical Group Co., Ltd., Shenyang, China). This section describes experimental design, sample preparation, data collection, and analysis methods.

### 4.1. Experimental Subjects and Conditions

Wild-type *Drosophila melanogaster* (Canton-S strain, stock number 9516, Indiana University, Bloomington, IN, USA) was used for the experiment. A total of 180 adult fruit flies were divided into three groups: experimental (2KGA-supplemented), positive control (ASA-supplemented), and negative control (no supplementation). Each group was further subdivided by sex (male and female), resulting in six subgroups (e.g., 9516-T-M for experimental group males, 9516-NC-F for negative control females), with 30 flies per subgroup. To ensure statistical reliability, each subgroup was distributed across three replicate test tubes, with 10 flies per tube. All flies were reared under controlled conditions (23 °C, 60% relative humidity, 12:12 h light:dark cycle) for 12 days.

### 4.2. Experimental Diets

The culture media was prepared using a standard cornmeal-yeast-agar recipe, with compositions detailed in Table 1. For the experimental group, 2KGA was added at 1.6 g/L. The positive control group received ASA at 1.62 g/L, while the negative control group received no supplementation. Distilled water was added to each medium to a final volume of 1 L. Maltose was hydrated before addition, and propionic acid was incorporated when the medium cooled to 60–70 °C to prevent degradation. All media were freshly prepared and stored at 4 °C in the dark to maintain stability.

### 4.3. HPLC Conditions

After loading the fruit flies into separate culture tubes, they were incubated for 12 days at a temperature of 23 degrees Celsius. Ten fruit flies from each group were taken and anesthetized using carbon dioxide and the samples were prepared as samples to be tested by grinding the samples with a grinder stick, followed by centrifugal extraction in preparation with L-ascorbic acid assay. It was dissolved in 0.3 mL of 1.0% metaphosphoric acid under bath condition. The homogenate was centrifuged for 10 min at room temperature, and the supernatant was taken and set aside. The supernatant was appropriately diluted with 1% metaphosphoric acid and filtered through a 0.22 micron injection filter. The filtered sample was injected into an AQ-C18 column for analysis. Water, acetonitrile and sodium phosphate were used to equip the mobile phase of the HPLC (Agilent 1100 Series system, Agilent Technologies, Santa Clara, CA, USA). Detection conditions: mobile phase, 20 mM sodium dihydrogen phosphate solution (pH 2.8):acetonitrile = 95:5; flow rate: 1.0 mL/min; temperature: 40 °C; detector: 243 nm. The ASA content of the samples was determined from the ASA standard curve. Subsequently, the ASA standard curve was plotted, and no less than five gradients of ASA standard solutions (0, 6.25, 12.5, 25, 50 μg/mL) were configured with pre-cooled 1% metaphosphoric acid solution, and the method of determination was the same as above. The ASA content in the samples was determined from the ASA standard curve.

## 5. Conclusions

In summary, this study demonstrates that exogenous 2-keto-L-gulonic acid (2KGA) supplementation significantly enhances L-ascorbic acid (ASA) content in *Drosophila melanogaster*. Rearing flies on 2KGA-supplemented media led to an approximately 25% increase in ASA levels relative to untreated controls, extending previous observations from plants and zebrafish to an invertebrate model. Sex-separated analyses further revealed that this effect is primarily driven by male flies, while females show a weaker or absent response, highlighting a pronounced sex-dependent regulation of ASA homeostasis in *Drosophila*. These findings provide the first experimental evidence that 2KGA can act as a metabolic precursor or regulator of ASA biosynthesis in an invertebrate and support the existence of endogenous, yet uncharacterized, vitamin C biosynthetic pathways in fruit flies. Given the central role of ASA in redox balance and stress resistance, *D. melanogaster* may serve as a convenient platform to dissect conserved mechanisms of redox regulation with relevance to human health. Future work should focus on identifying the enzymatic steps involved, integrating transcriptomic and genetic approaches, and establishing dose–response relationships across multiple 2KGA concentrations to refine and extend the present proof-of-concept.

## Figures and Tables

**Table 1:** Composition of Culture Media for Each Experimental Group.

Group	Negative Control (NC)	Treatment (T)	Positive Control (PC)
Sucrose (g/L)	40.04	40.04	40.04
Soy flour (g/L)	9.16	9.16	9.16
Maltose (g/L)	42.21	42.21	42.21
Agar (g/L)	10.00	10.00	10.00
Sodium benzoate (g/L)	1.00	1.00	1.00
Methylparaben (g/L)	0.27	0.27	0.27
Cornstarch (g/L)	66.25	66.25	66.25
Propionic acid (mL/L)	6.875	6.875	6.875
2-Keto-L-Gluonic Acid (g/L)	N/A	1.60	N/A
L-Ascorbic Acid (g/L)	N/A	N/A	1.62

## Data Availability

The original contributions presented in this study are included in the article. Further inquiries can be directed to the corresponding author.
